# Death Following the Operation for Congenital Cataract

**Published:** 1835

**Authors:** 


### II.—Death following the operation for Congenital Cataract.

In the year 1834, a little girl, five or six years old, was brought from Indiana to the *Cincinnati Eye Infirmary*, with congenital cataract. She was apparently of infirm constitution, and but little developed in her intellectual functions. The needle, for the performance of Mr. Sanders' operation, was introduced through the cornea, and, as the pupil was broadly dilated, the iris was not touched. A slight laceration of the capsule of the lens of each eye, was followed by its breaking up, and the immediate appearance of a black color. Thus the operation was attended with the least possible violence. Nevertheless, she very soon fell into a deep state of nervous irritation, which involved the brain, and in a few days proved fatal.

